# Elucidation of Factors Affecting Anterior Occlusion in Primary Dentition Based on the Japan Environment and Children’s Study

**DOI:** 10.3390/children12020254

**Published:** 2025-02-19

**Authors:** Risa Ishiko, Kotaro Sena, Ichie Koseki, Masumi Sasai, Chiharu Ota, Takeyoshi Koseki

**Affiliations:** 1 Department of Oral Supportive Care and Management, Tohoku University Hospital, Sendai 980-8575, Japan; risa.ishiko.e4@tohoku.ac.jp; 2 Department of Community Social Dentistry, Division of Preventive Dentistry, Tohoku University Graduate School of Dentistry, Sendai 980-8575, Japan; ichie.koseki.c1@tohoku.ac.jp (I.K.); sasai.masumi.r6@dc.tohoku.ac.jp (M.S.); takeyoshi.koseki.b6@tohoku.ac.jp (T.K.); 3 Department of Development and Environmental Medicine, Tohoku University Graduate School of Medicine, Sendai 980-8575, Japan; chiharu.ota.e8@tohoku.ac.jp

**Keywords:** cohort study, malocclusion, dentition, primary, open bite, anterior occlusion

## Abstract

**Background/Objectives**: Malocclusion in primary dentition affects permanent dentition. However, the factors contributing to malocclusion in the oral cavities of children have not been fully elucidated. We hypothesized that environmental factors affect malocclusion in the primary dentition of the Japanese population and aimed to identify factors associated with anterior occlusion in primary dentition. **Methods**: The study involved 3793 parent–child pairs from the Miyagi Regional Centre as part of a supplementary survey to the Japan Environment and Children’s Study, a cohort study. A questionnaire assessing oral development and environmental factors was administered to parent-child pairs who consented to participate. Parents assessed anterior occlusion when their children were 3.5 years old. **Results**: The maxillary primary central incisors tended to erupt earlier in the open bite group. Significantly more children in this group were breastfed until 1 year and drank ionic beverages at 1.5 years. In addition, thumb sucking or pacifier use was significantly common at 2 years of age. A correlation was observed between the mother’s body mass index (BMI) before and after pregnancy and anterior occlusion. **Conclusions**: In the open bite group, the occlusion status of the anterior teeth at 3.5 years of age showed distinctive results influenced by the eruption period of the primary anterior teeth, oral habits, intake of sweetened beverages, and maternal BMI. These findings suggest that parental observation could be useful for screening children for malocclusion until the eruption of permanent dentition.

## 1. Introduction

Oral nutrition is important to nurture healthy growth and maintain good health and oral function in infants and toddlers. Malocclusion affects bone morphology [1] and respiratory and chewing functions [2,3]. Therefore, good occlusal conditions are necessary to maintain good masticatory function and ensure a healthy oral environment for oral intake [4]. The occlusal state is closely related to the position and direction of tooth eruption, and the timing is dependent on age [5,6,7,8], nutrition, lifestyle habits, and environmental changes [6,7,8].

Generally, the central maxillary and mandibular incisors erupt first between the ages of 8 and 12 months [6,7,8,9]. Continuing the sequential teething, the second molars erupt between 23 and 33 months; primary dentition is completed at approximately 2.5 years of age. One year later, at the age of 3.5 years, masticatory function is stable, and speech function is fully developed [6,7,8,9].

Simultaneously, maxillary growth is considered an intermediate type of neural and general growth in Scammon’s Growth Curve. The end of the first growth phase for height [10] is considered to be around age 6; therefore, the growth curve at 3.5 years corresponds to the latter part of a period of rapid development. By the age of 6, palatal width, height, and length increase by over 90% of their size at birth.

Malocclusion in the primary dentition affects the permanent dentition [11,12,13,14]. Hence, maintaining proper primary occlusion is fundamental for lifelong good oral health. Various factors are believed to contribute to malocclusion, including discrepancies in the size of the teeth and jawbone, imbalances in the growth rate of the jawbone, inappropriate oral habits (such as thumb sucking, tongue habits, and mouth breathing), and congenital or acquired tooth loss [15,16,17]. Some malocclusions in the primary dentition may improve with growth as the permanent dentition replaces the primary dentition and the jawbone grows and develops [18]. However, the factors contributing to malocclusion in the primary dentition remain unclear. These factors are intricately intertwined, making the assessment of the malocclusion risk a challenge [15,19]. Malocclusion is a common problem globally despite regional differences in prevalence and types of malocclusions [20]. Regional differences may be due to socioeconomic status, lifestyle (such as diet and cultural habits), genetics, and environmental factors, such as exposure to chemicals. A large-scale survey in Japan may help characterize the occlusal status of the Asian population.

The Japan Environment and Children’s Survey (JECS) is a prospective birth cohort study targeting approximately 100,000 children and their parents from conception to the age of 13 years across Japan [7,21,22]. The goal of JECS is to understand how the environment affects children’s health, and pertinent to the current study, additional questionnaires regarding oral development, including the occlusal status and eruption period of the deciduous anterior teeth, were administered [7]. We hypothesized that environmental factors may influence malocclusion in the primary dentition of the Japanese population. Therefore, by using JECS datasets, we aimed to identify environmental factors associated with anterior occlusion in the primary dentition at age 3.5 when masticatory function is still developing.

## 2. Materials and Methods

### 2.1. Study Population

**Figure 1:**
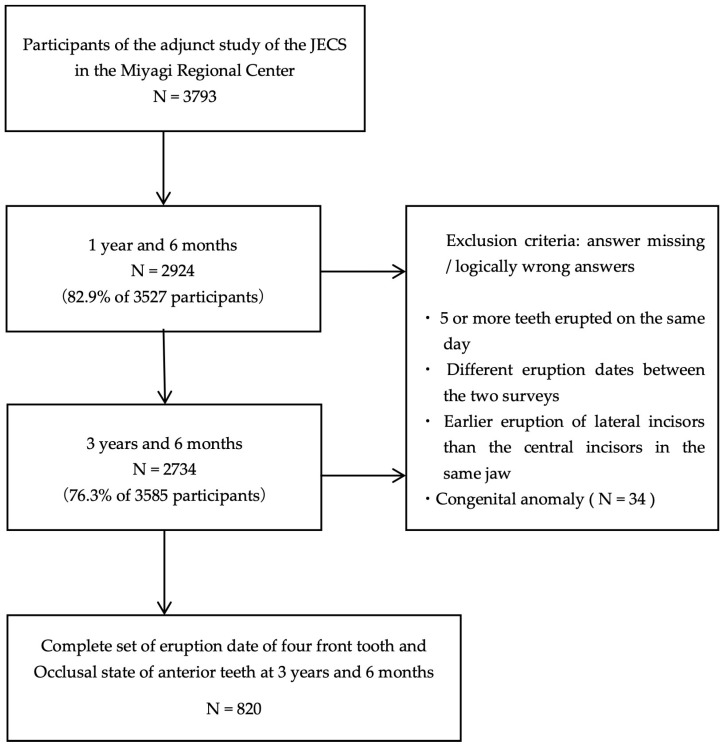
Flow diagram of enrollment of participants in the Japan Environment and Children’s Study (JECS) adjunct study in the Miyagi Regional Centre.

This study is an adjunct of the JECS conducted at the Miyagi Regional Centre. The study design of the JECS has been described previously [21]. Briefly, pregnant women were recruited from 15 regional centers, including the Miyagi Regional Centre, between January 2011 and March 2014. We included the following participants: (1) those residing in the study areas at the time of the recruitment and expected to reside continually in Japan for the foreseeable future, (2) those with an expected delivery date between 1 August 2011 and mid-2014, and (3) those able to participate in the study without difficulty, that is, they must be able to comprehend the Japanese language and complete the self-administered questionnaire. At the Miyagi Regional Centre, 9217 participants were registered during this period across 14 municipalities in Miyagi Prefecture. Of the 9217 participants, 3793 agreed to participate in the study, and written informed consent was obtained from all participants. Of the 3793 participants who agreed to participate in the supplementary study, 2924 and 2734 responded to the 1.5- and 3.5-year surveys, respectively. After excluding responses that met the exclusion criteria, 820 valid responses were obtained (Figure 1) from 416 male and 404 female participants. The exclusion criteria were missing and/or logically inconsistent responses. Examples of logically inconsistent answers included the eruption of five or more teeth on the same day, different eruption dates between the two surveys, and the earlier eruption of the primary lateral incisors compared to the primary central incisors in the same jaw. In addition, responses from participants with congenital anomalies were excluded (Figure 1).

### 2.2. Methods

A Japanese questionnaire comprising surveys for socioeconomic status (for example, education, employment, household income, social capital, and community support), lifestyle factors (stress levels, diet, smoking and alcohol habits, physical exercise activities, sleep, infections, and medications), and physical environment (heat, ionizing radiation, housing condition, and neighborhood) were self-administered, and no assistance was provided, except for sample answers provided with the questionnaire. The questionnaire was administered twice between 2013 and 2016 at 1.5 and 3.5 years of age. A diagram of the dentition was included in the questionnaire, and the respondents were asked to indicate the month and date of the eruption of the primary teeth, such as the maxillary and/or mandibular primary central incisors and maxillary and/or mandibular primary lateral incisors [7].

**Figure 2:**
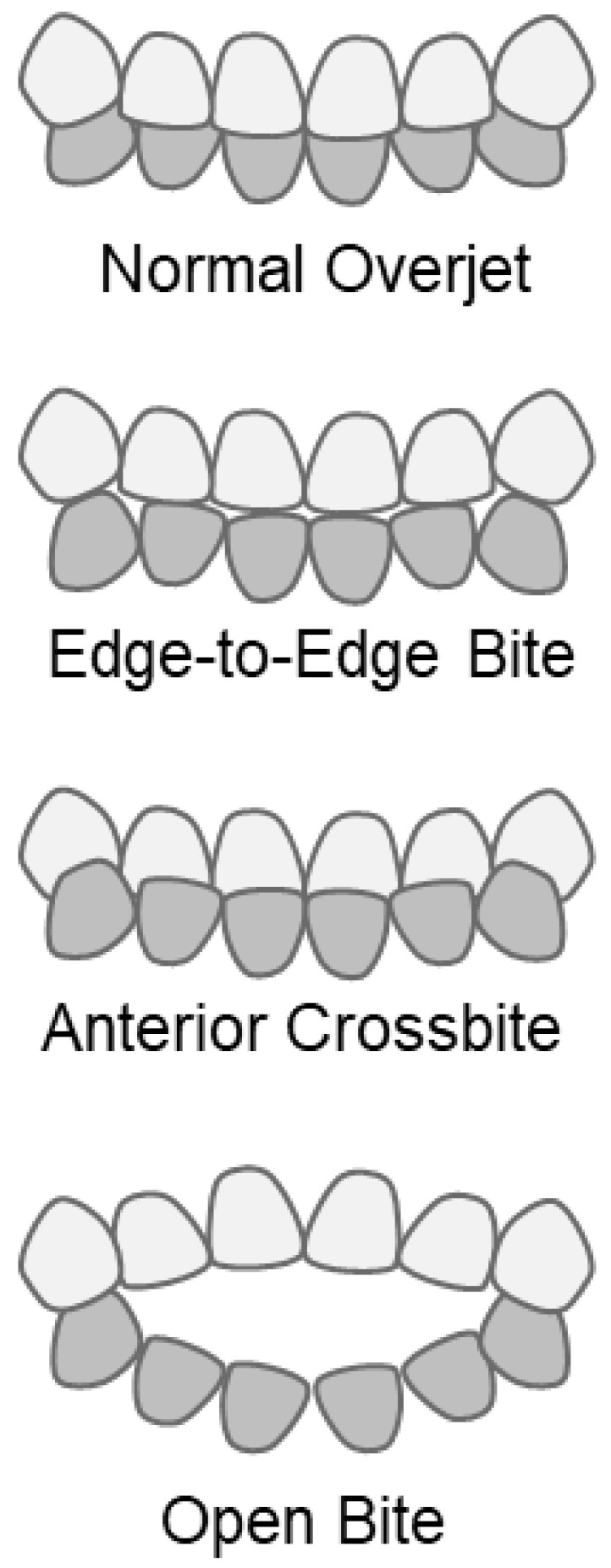
Questionnaire on occlusal state at 3.5 years.

The respondents were asked to fill in the occlusal status of their anterior teeth at 3.5 years of age. The occlusal status was classified into four categories (Figure 2).

The respondents were asked to choose one of the four occlusal conditions: (i) normal overjet, a state in which the upper teeth protrude in front of the lower teeth; (ii) edge-to-edge bite, a state in which the tips of the upper and lower teeth are perfectly aligned; (iii) anterior crossbite, a state that the lower teeth are placed above the upper teeth; (iv) open bite, a condition where there is a gap between the upper and lower teeth.

### 2.3. Statistical Analysis

Statistical analysis was performed using JMP version 17.2.0 (SAS Institute Inc., Cary, NC, USA). The Chi-Square test was used to analyze the distribution of participants’ occlusal statuses. The Kruskal–Wallis test was used to examine the relationship between the eruption period of deciduous incisors and occlusal status, as well as between maternal body mass index (BMI) and occlusal status. The significance level was set at *p* < 0.05.

## 3. Results

### 3.1. Anterior Occlusion of Study Participants

The distribution of the participants’ anterior occlusions is shown in Table 1. The most common anterior occlusion was normal (normal overjet group), which included 516 participants (62.9%) consisting of 257 male and 259 female participants. The edge-to-edge bite group included 229 participants (27.9%) comprising 124 male and 105 female participants, and 60 participants (7.3%) were included in the anterior crossbite group (26 male and 34 female participants). The least common anterior occlusion was in the open bite group, which included 15 participants (1.8%) comprising 9 male and 6 female participants. No significant differences were observed between the sexes in any of the anterior occlusions.

### 3.2. Relationship Between Anterior Occlusion and Eruption Order/Period

The participants were classified according to the order of front teeth eruption (primary central incisors and primary lateral incisors) in the maxillary and mandibular bones (Table 2). The most common eruption order was mandibular primary central incisor, followed by maxillary primary central incisor, maxillary primary lateral incisor, and mandibular primary lateral incisor, and this was observed in 187 participants (22.8%). However, no significant relationship was found between eruption order and anterior occlusal status.

**Figure 3:**
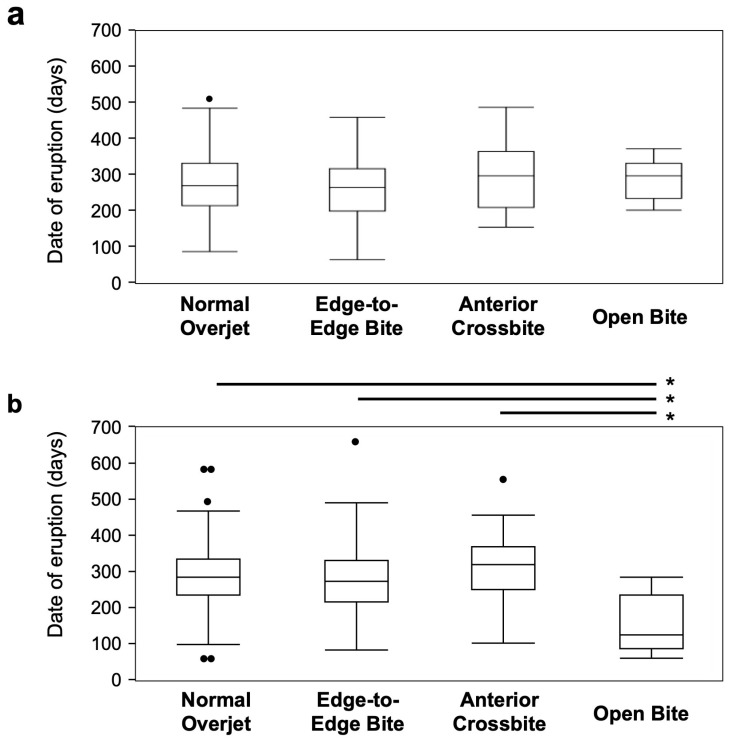
Analysis of the relationship between occlusal status and eruption period of front teeth. No significant differences were observed between the male (**a**) and female (**b**) participants. In female participants, the eruption period of the maxillary primary central incisors was significantly earlier in the open bite group compared with the other groups. The black dots indicate outliers. *: *p* values < 0.05.

Next, when analyzing the relationship between anterior occlusion and the eruption period of primary incisors, no significant differences were observed in male participants (Figure 3a). In contrast, in female participants, the eruption period of maxillary primary central incisors was significantly earlier (*p* values = 0.006–0.011) in the open bite group compared with the other occlusal status groups (Figure 3b).

### 3.3. Relationship Between Occlusal Status and Lifestyle Habits

**Figure 4:**
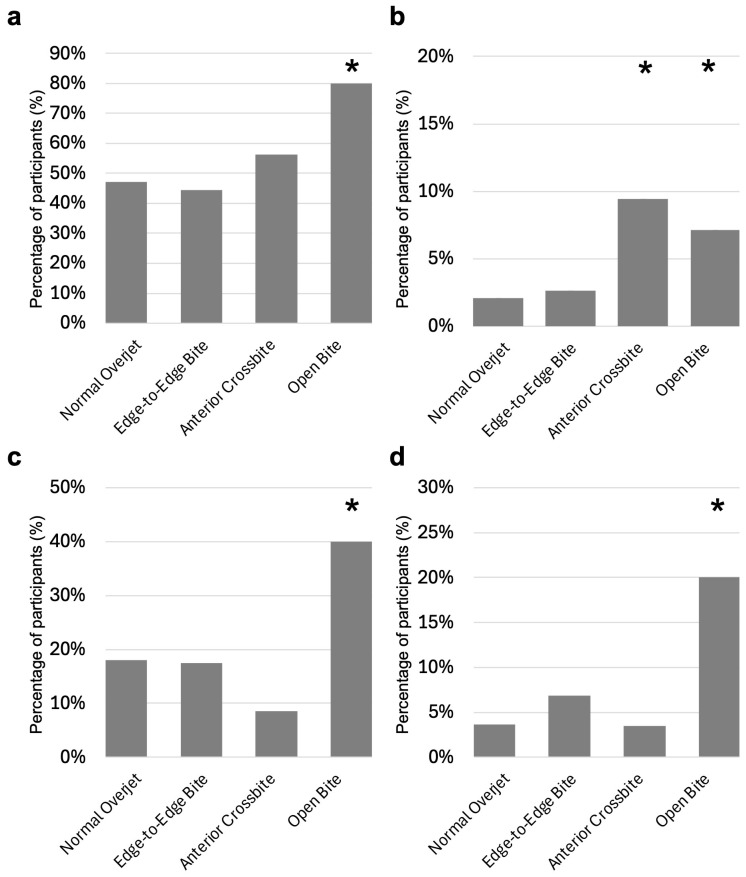
Analysis of the relationship between occlusal status and lifestyle habits. (**a**): Breastfeeding at 1 year of age. (**b**): Drinking ionic beverages at 1.5 years of age. (**c**): Thumb sucking at 2 years of age. (**d**): Pacifier use at 2 years of age. *: *p* values < 0.05.

To clarify the lifestyle habits influencing the occlusal status of the primary anterior teeth, we analyzed the relationship between anterior occlusal status and the lifestyle habits included in the questionnaire. In the open bite group, breastfeeding at 1 year of age, drinking ionic beverages at 1.5 years of age, and thumb sucking and pacifier use at 2 years of age were significantly more common compared with other occlusal status groups (Figure 4). Compared with other occlusal status groups, drinking ionic beverages at 1.5 years of age was significantly more prevalent in the anterior crossbite group.

Breastfeeding at 1 year of age and drinking ionic beverages at 1.5 years of age were significantly more common in the open bite group than in the other occlusal status groups (*p* values < 0.05). In addition, thumb sucking and pacifier use at 2 years of age were significantly more common in the open bite group than in the other occlusal status groups (*p* values < 0.05).

### 3.4. Relationship Between Occlusal Status and Maternal BMI

**Figure 5:**
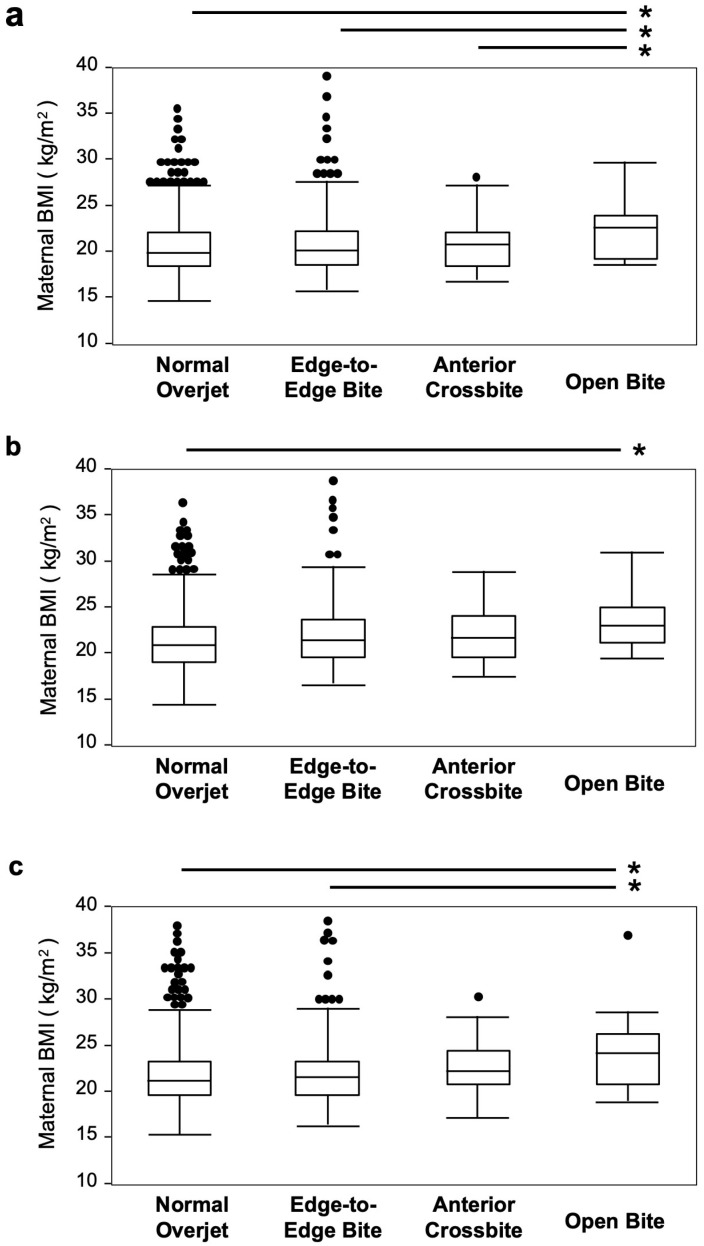
Analysis of the relationship between occlusal status and maternal BMI. Maternal BMI at (**a**): early pregnancy. (**b**): 6 months after childbirth. (**c**): 2.5 years after childbirth. BMI: body mass index. The black dots indicate outliers. *: *p* values < 0.05.

We analyzed the influence of maternal BMI on the participants’ occlusal status (Figure 5). Maternal BMI was significantly higher in the open bite group compared with the normal overjet group from before pregnancy until 2.5 years after childbirth (*p* values = 0.011–0.028). During early pregnancy, the maternal BMI was significantly higher in the open bite group than in the normal overjet group, as well as in the edge-to-edge bite and anterior crossbite groups (*p* values = 0.011–0.044).

In the open bite group, maternal BMI was significantly higher before pregnancy compared with other occlusal status groups. At 6 months after childbirth, the maternal BMI was significantly higher than that in the normal overjet group. At 2.5 years after childbirth, it was significantly higher than that in the normal overjet and edge-to-edge bite groups.

## 4. Discussion

In this study, we analyzed the occlusion status of the anterior teeth at 3.5 years of age (which can be observed by parents), lifestyle habits, and environmental factors that affect occlusion. We found that the period of eruption of the primary anterior teeth, lifestyle habits, and maternal BMI had a potential influence on the primary anterior open bite. These results partially support our hypothesis that environmental factors may influence malocclusion in the primary dentition of the Japanese population.

In general, occlusion status is diagnosed by dentists and classified into detailed categories, such as normal occlusion, incisal occlusion, reverse occlusion, open bite, crossbite, overbite, and maxillary protrusion [23,24]. However, this study relied on parents’ intraoral observations, making it difficult to conduct more advanced and specialized oral observations. Therefore, we focused on the occlusal condition of the front teeth, which can be easily assessed without specialized knowledge. We divided these into four categories: normal occlusion (normal overjet group), incisal occlusion (edge-to-edge bite group), reverse occlusion (anterior crossbite group), and open bite (open bite group).

A systematic analysis by Chen et al. showed that the global prevalence of anterior crossbite in primary dentition was 7.8%; that of open bite was not calculated due to the large variation in the definitions, measurements, and methodologies, but was reported to range from 0.7–15% in 13 Asian studies [20]. Ridder et al. reported a 7.35% prevalence of anterior crossbite and a 4.71% prevalence of anterior open bite in Asia [25]. In this study, the prevalence of anterior crossbite was 7.3%, and that of anterior open bite was 1.8%. These results are similar to the systematic reports and indicate that parental observation can be useful for screening for malocclusion in children.

It has been determined that these malocclusions are caused by oral functional abnormalities and may not necessarily require therapeutic intervention [19,26]. If these abnormalities persist after the permanent teeth have erupted and the jawbone has grown, the effects will likely persist [16,27]. In addition, improvement is less likely in cases of anterior crossbites and open bites. Oral habits such as daily pacifier use, which is related to the thumb/digit sucking [28], as well as tongue thrusting and mouth breathing [29], influence malocclusion. Barrera et al. also reported significant relationships in the vertical plane with atypical swallowing and lip sucking and in the horizontal plane with oral breathing, atypical swallowing, and digit sucking [27].

The most well-known factors causing functional abnormalities are thumb sucking, nail biting, and the use of pacifiers [17,18,30]. It is believed that holding a finger or pacifier between the upper and lower primary incisors for an extended period pushes these teeth apart, leading to an open bite [28]. This study’s findings also suggest that thumb sucking and pacifier use in 2-year-old children are contributing factors for open bites, which is consistent with findings from previous studies.

Teeth alignment is maintained by the balance between tongue pressure [31] and the muscles around the mouth [30]. The weaning period coincides with the eruption of the primary incisors, a time when significant changes occur in perioral movements, including swallowing patterns [32,33]. During the early weaning period, the tongue moves back and forth, and the lower lip tends to enter the oral cavity. During the middle weaning period, the tongue begins to move up and down, pressing against the upper palate to crush food. If the maxillary primary central incisors erupt before or during weaning, the lower lip and tongue may continue to press forward and upward on these teeth for extended periods. Consequently, the maxillary primary central incisors are forced forward and upward, leading to an anterior open bite. This study’s findings suggest that the eruption of the maxillary primary central incisors may be weakly associated with open bites.

The association between anterior open bite and atypical swallowing has been reported [34]. Inchingolo et al. reported that atypical swallowing occurs when there is no transition from infantile to adult swallowing. In atypical swallowing, the tongue’s posture is altered with the tongue’s tip touching the front, palatal surface, or between the dental arches rather than the palate. This tongue-pushing or tongue-thrust habit is thought to induce open bites [35]. In our study, breastfeeding at 1 year of age was found to be related to open bites, suggesting that the duration of breastfeeding may be a risk factor for open bites [36]. In contrast, if the lip-closure force is weak, it leads to lip incompetence and can exacerbate anterior malocclusion. Mouth breathing has also been reported to increase the risk of open bites [37]. Further research is needed to understand the mechanism causing malocclusion in children.

Due to the extreme heat in summer caused by abnormal weather in recent years, the risk of heat stroke and dehydration has increased, and thus, the consumption of ionic beverages has also been on the rise. In the open bite group, significantly more children drank ionic beverages at 1.5 years of age. When ionic beverages are consumed in large quantities, the required intake of vitamin B1 increases more than usual due to the large amount of sugar intake [38]. Vitamin B1 deficiency can lead to edema, loss of appetite, vomiting, neurological symptoms, muscle weakness, impaired consciousness, severe heart failure, encephalopathy [39,40], and weakness of the perioral muscles.

Further, frequent consumption of large amounts of sugary soft drinks can lead to a condition known as “soft drink ketosis” [41,42]. When large amounts of sweet drinks are consumed, rapid elevation of blood sugar levels leads to general fatigue, drowsiness, frequent urination, dry throat, and, in severe cases, coma. This condition is particularly common in young people in their teens to 30s, and persons who tend to be obese are particularly at risk. Ketoacidosis causes dry throat, which, in turn, leads to increased consumption of soft drinks to alleviate thirst, thereby creating a vicious cycle that exacerbates the ketoacidosis condition. This often results in mouth breathing, which may increase the risk of anterior malocclusion, particularly an open bite. Based on the above, daily consumption of ionized beverages might contribute to an open bite through declining perioral muscles and mouth breathing.

Regarding maternal factors, the maternal BMI before pregnancy, 6 months post-partum, and 2.5 years post-partum were significantly higher in the open bite group than in the other groups. This may be related to the possibility that the mother also had an open bite. Skeletal open bites have been suggested to be influenced by genetic factors [19]. In addition, fewer teeth had occlusal contact in individuals with open bites compared with those with normal occlusions. Therefore, the maximum occlusal force and chewing efficiency in individuals with open bites are lower than those in individuals with normal occlusion [43,44]. Reduced chewing efficiency is associated with metabolic syndrome and may contribute to obesity [45]. The mother’s lifestyle habits related to the intake of sweet drinks might reflect in the child’s diet and may lead to open bites from consumption of the aforementioned sweet drinks. If a mother has an open bite, she may have lower chewing efficiency and a greater tendency toward obesity compared with mothers with normal occlusion. This may explain the significantly higher BMI observed in this study. However, as the mother’s occlusal condition was not evaluated in this study, this hypothesis was difficult to verify. Future research should include assessments of the mother’s occlusal condition to enable more comprehensive analyses and evaluations.

Recent reports outside Japan have identified non-nutritional sucking habits [46], breastfeeding duration [47,48], mouth breathing [49], and socioeconomic status [50] as environmental factors that influence malocclusion during the primary dentition stage. Non-nutritional sucking habits, such as pacifier use and thumb sucking, were also observed in the present study. However, to the best of our knowledge, the influence of ionic beverage intake or mothers’ BMI is yet to be reported.

The strength of this study lies in its large-scale and long-term cohort design. However, several limitations should be acknowledged. First, the occlusion status was not confirmed by dental professionals. Second, data on the occlusal conditions of the mothers and fathers were not available. Third, data for the period after tooth replacement were missing. Therefore, future studies should analyze the influence of environmental factors longitudinally by following up with the cohort to the permanent dentition period beyond the tooth replacement stage. Furthermore, the participants’ genetic information should be analyzed since genetic factors are known to influence the types of malocclusions.

## 5. Conclusions

This study aimed to identify factors associated with anterior occlusion in primary dentition based on the questionnaire survey at the Miyagi Regional Centre as a supplementary survey to the JECS. The results showed that the eruption period of the primary anterior teeth, oral habits, intake of ionic beverages, and maternal BMI influenced the open bite group at age 3.5 years. The current results suggest the potential usefulness of parental observation in screening children for malocclusion until the eruption of permanent dentition.

## Figures and Tables

**Table 1:** Distribution of participants’ occlusal status.

	Total		Male		Female	
All	820	100.0%	416	100.0%	404	100.0%
Normal overjet	516	62.9%	257	61.8%	259	64.1%
Edge-to-edge bite	229	27.9%	124	29.8%	105	26.0%
Anterior crossbite	60	7.3%	26	6.3%	34	8.4%
Open bite	15	1.8%	9	2.2%	6	1.5%

**Table 2:** Eruption order of the primary front teeth.

Eruption Order of Front Teeth	Total		Normal Overjet		Edge-to-Edge Bite		Anterior Crossbite		Open Bite	
(LA ^§^) - (UA ^†^) - (UB ^‡^) - (LB ^¶^)	187	22.8%	132	25.6%	42	18.3%	7	11.7%	6	40.0%
(LA) - (UA) - (LB) - (UB)	99	12.1%	65	12.6%	24	10.5%	9	15.0%	1	6.7%
(LA) - (UA) - (UB) = (LB)	36	4.4%	19	3.7%	12	5.2%	4	6.7%	1	6.7%
(LA) - (UA) = (UB) - (LB)	20	2.4%	12	2.3%	8	3.5%	0	0.0%	0	0.0%
(UA) = (LA) - (UB) = (LB)	33	4.0%	17	3.3%	13	5.7%	2	3.3%	1	6.7%
(UA) = (LA) - (UB) - (LB)	5	0.6%	4	0.8%	1	0.4%	0	0.0%	0	0.0%
(LA) - (UA) = (LB) - (UB)	4	0.5%	2	0.4%	0	0.0%	2	3.3%	0	0.0%
(LA) - (LB) - (UA) - (UB)	25	3.0%	18	3.5%	5	2.2%	2	3.3%	0	0.0%
(UA) - (LA) - (UB) - (LB)	19	2.3%	10	1.9%	7	3.1%	1	1.7%	1	6.7%
(UA) - (UB) - (LA) - (LB)	9	1.1%	5	1.0%	4	1.7%	0	0.0%	0	0.0%
Others	383	46.7%	232	45.0%	113	49.3%	33	55.0%	5	33.3%
Total	820	100.0%	516	100.0%	229	100.0%	60	100.0%	15	100.0%

Eruption order in front teeth: -, sequential eruption; =, eruption in same time; ^†^ UA, maxillary primary central incisors; ^‡^ UB, maxillary primary lateral incisors; ^§^ LA, mandibular primary central incisors; ^¶^ LB, mandibular primary lateral incisors. (χ^2^ test, *p* = 0.172).

## Data Availability

The original contributions presented in the study are included in the article, further inquiries can be directed to the corresponding author.

## Abbreviations

The following abbreviations are used in this manuscript:

BMI	Body mass index
JCES	Japan Environment and Children’s Survey
